# MicroRNA-183 in Cancer Progression

**DOI:** 10.7150/jca.39044

**Published:** 2020-01-01

**Authors:** Dingren Cao, Min Di, Jingjie Liang, Shuang Shi, Qiang Tan, Zhengguang Wang

**Affiliations:** 1 College of Animal Sciences, Zhejiang University, Hangzhou, 310058, P. R. China.; 2 Sir Run Shaw Hospital, Zhejiang University College of Medicine, Hangzhou, 310058, P. R. China.

**Keywords:** microRNA-183, cancer progression, EMT, metastasis, microangiogenesis

## Abstract

MicroRNA-183(miR-183) is abnormally expressed in many kinds of tumors. It participates in the initiation and development of tumors. There are many pathways regulate the expression of miR-183. The action mechanism of miR-183 in cancer is very extensive, and contradictory conclusions are often drawn. It was upregulated in 18 kinds of cancer, downregulated in 6 kinds of cancer. In addition, there are seven types of cancer, both upregulated and downregulated reports can be found. Evidence showed that miR-183 can not only directly play the role of oncogene or antioncogene, but also regulate the expression of other oncogene or antioncogene in different cancer types. In this review, we discuss the regulator of miR-183 and summarized the expression of miR-183 in different cancers. We also counted the target genes of miR-183 and the functional roles they play. Furthermore, we focused on the roles of miR-183 in cell migration, cell invasion, epithelial-mesenchymal transition (EMT) and microangiogenesis, which play the most important roles in cancer processes. It sheds light on the likely reasons why miR-183 plays different roles in various cancers. In addition, miR-183 and its downstream effectors have the potential to be promising prognostic markers and therapeutic targets in cancer.

## Introduction

Mature microRNA (miRNA) is a kind of small noncoding single stranded RNA with 18 to 24 nucleotides 1. The latest release of miRBase (v22) contains miRNA sequences from 271 organisms: 38 589 hairpin precursors and 48 860 mature miRNAs. Human genome contains 1917 annotated hairpin precursors, and 2654 mature sequences 2. MiRNAs regulate the expression of target genes by completely or incompletely complementing the 3'UTR untranslated region of target miRNAs. A single miRNA can be associated with several or even hundreds of target genes. A gene is regulated by multiple miRNAs at the same time 3. MiRNAs are ubiquitous in eukaryotic cells and highly conserved among different species. The expression of miRNAs has obvious sequence specificity and tissue specificity 4.

MiR-183 is a member of the microRNA-183-96-182 cluster located at the 7q31-34 locus of human chromosome 5. It's highly conserved in different organisms. The expression of miR-183 is tissue-specific and highly enriched in visual and sensory organs. It participates in the development of retina and cochlea and plays an important role in maintaining normal function 6.

In recent years, many studies have shown that miR-183 is abnormally expressed in many kinds of tumors, and participates in the initiation and development of tumors. However, in different cancer tissues and cell lines, there are great differences in the expression of miR-183. Even in the same type of cancer, opposite expression results are often reported. While in some studies, the expression of miR-183 was inconsistent in different samples of the same pathological tissue. Therefore, it is urgent to summarize the expression of miR-183 in different types of cancer and function targeted by miR-183. All previous published articles reviewed the three members of miR-183 family together, but the three members of miR-183 family, miR-183, miR-182 and miR-96, differ greatly both in upstream regulators and target genes. It is easy to conceal the function of miR-183 when review the family together. This article is focusing only on miR-183 in cancer progression. The regulatory ways of miR-183 from upstream to downstream regulation are systematically and comprehensively elaborated. In this review, we summarized the expression of miR-183 in different cancers, counted the target genes of miR-183, and focused on the role of miR-183 in cell migration, cell invasion, EMT and microangiogenesis, which play the most important roles in cancer processes. The latest research of miR-183 in lung cancer, ovarian cancer, bladder cancer and prostate cancer has also been fully reflected in this paper. This review provides a basis for elucidating the role of miR-183 in cancer progression.

## Regulation of miR-183 expression

The expression of miR-183 varies greatly among different types of cancer. So it is necessary to summarize the upstream regulatory factors of miR-183 in order to clarify the different up-and-down regulation phenomena of miR-183 in different pathological tissues. The upstream regulation mechanism of miR-183 cluster has been reported frequently, but only a part of the factors regulate the expression of miR-183 directly.

The reported literature shows that there are eight regulatory factors that can upregulate miR-183 expression level. They can promote the transcriptional expression of miR-183 by binding to the upstream promoter region. The regulation of Wnt/beta-catenin (CTNNB1) signaling pathway on miR-183 is the most studied. Activation of CTNNB1 can upregulate the expression of miR-183, knockdown of CTNNB1 also showed concordant downregulations of miR-183, suggesting that miR-183 may be an important downstream target gene in the regulation of Wnt signaling pathway 7. Other studies have shown that expression of GSK3β can inhibit the expression of miR-183 through the β-Catenin/TCF/LEF-1 pathway in human gastric cancer cells, while β-Catenin/TCF/LEF-1 binds to the promoter of miR-183 and thereby activates the transcription of miR-183 8. While in non-small cell lung cancer, they found that TFAP2C blocked AKAP12-mediated cyclin D1 inhibition by inducing the overexpression of miR-183, which shows that TFAP2C is one of the upstream regulators of miR-183 9. In breast cancer, the transcription of miR-183 was regulated by ZEB1 and HSF2, HSF2 can upregulate miR-183 expression 10. While the regulatory effect of ZEB1 on miR-183 is more complex. ZEB1 can downregulate the transcriptional expression of miR-183, conversely, ZEB1 can be targeted by miR-183. In addition, there are other regulatory factors can downregulate the transcriptional expression of miR-183 by binding to the upstream promoter region of miR-183. Studies in neuroblastoma show that HDAC(s) involved in miR-183 transcriptional and downregulated it. MYCN was found to recruit HDAC2 in the same complexes to the miR-183 promoter 11. What's more, inhibition of PI3K/AKT signaling pathway also significantly reduced the expression of miR-183 in esophageal squamous cell carcinoma (ESCC) cells 12.

**Figure 1:**
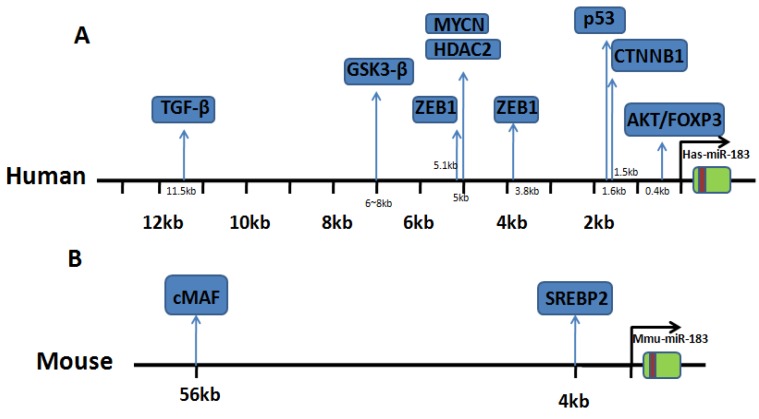
** Binding site location of transcriptional regulators.** (A) Research in human genome: binding sites of transcriptional factor in upstream of miR-183. (B) Research in mouse genome: binding sites of transcriptional factor in upstream of miR-183.

IncRNA plays an important role in regulating the expression of miRNA. IncRNA MALAT1 function is a sponge competitive endogenous RNA for miR-183 that inhibits the expression and function of miR-183 13. The above literature indicates the complexity of the regulation mechanism of miR-183 expression in different cells. We summarize the upstream regulating direction of miR-183 and the binding site location. Details are shown in Table 1 and Fig. 1.

## Dysregulation of miR-183 in different cancer

Upstream of miR-183 is regulated by a variety of regulatory factors. In different cancers, different transcription factors play a major role and expression of miR-183 varies greatly (Table 2). Results showed that miR-183 was upregulated in 18 kinds of cancer including prostate cancer, ovarian cancer, breast cancer, et al, down regulated in 6 kinds of cancer including osteosarcoma, melanoma, et al. In these cancer cases, miR-183 upregulated and plays an oncogene role while plays as an antioncogene when downregulated. But in 7 kinds of cancer like hepatocellular cancer (HCC), endometrial cancer, some research reported that miR-183 was upregulated in tissues or cell lines, while others reported that miR-183 was downregulated (Table 2). MiR-183 can play a dual role in promoting or inhibiting those cancers. In HCC, Li et al. found that among the 25 samples analyzed, miR-183 was significantly upregulated (twofold to 367-fold) in 17 samples compared with the matching nontumoral liver tissues 14. Studies in breast cancer have shown similar results 33. This indicates that miR-183 expression has strong tissue specificity and individual differences. The wide variability in the reported results may be attributable to the differences between blood and tissue or the heterogeneity in cancer cells.

## MiR-183 in cancer progression

MiR-183 can play the role of both oncogene and antioncogene through act on different genes among all kinds of cancers. We statistical analyze target genes of miR-183, which have been identified. These target genes are mainly function in EMT, migration, invasion and microangiogenesis. These aspects play a key role in the process of cancer, which directly reflects that miR-183 is a key role in the process of cancer.

### Roles of miR-183 in EMT

The malignant progression of tumors is mainly manifested in the enhancement of the ability of migration and invasion. While the first step for cell invasion and migration is to change from epithelioid to mesenchymal 15. EMT refers to the change of epithelial cells, which lose polarity, tight junction and adhesion between cells, acquire the ability of infiltration and migration, and become mesenchymal cells with morphology and characteristics 16. In recent years, the research of EMT has turned to miRNAs. Results show that miR-183 is involved in many aspects of EMT, including apoptosis, tissue repair, extracellular matrix remodeling, and metastasis of tumors 17.

In malignant epithelial cells, ZEB1 acts as an activator of EMT, triggering the phenotype transformation of epithelial cells to mesenchymal cells 18. Studies found that miR-183 was able to target ZEB1 19. MiR-183 represses EMT through the regulating of ZEB1, overexpression of miR-183 results in the change of cells from mesenchymal to epithelial. E-cadherin is an EMT associated marker. ZEB1 can bind with E-box in E-cadherin promoter to inhibit E-cadherin expression 20. Several clinical studies have shown that increasing the expression of E-cadherin can significantly improve life quality and prolong the survival time of cancer patients with several types of tumors 21. Overexpression of miR-183 can downregulate E-cadherin expression and increase N-cadherin expression 22, 23.

Metastasis-associated 1 (MTA1) protein is a prime inducer of EMT and metastatic progression in all solid tumors. MTA1 protein and RNA expression showed opposite trends to miR-183 expression in breast, renal, prostate, and testicular cancer tissues. Sun et al. found that in osteosarcoma tissues and MG63 cells, miR-183 inhibits EMT and apoptosis by targeting MTA1 24. In nasopharyngeal carcinoma spheroids, ectopic expression of miR-183 markedly suppressed EMT and strikingly enhanced DDP-induced apoptosis, whereas MTA1 overexpression partially reversed these effects 25. In human NSCLC cells, miR-183 downregulates MTA1 to inhibit the proliferation and EMT. In pancreatic cancer, Lin et al. found that overexpression miR-183 decreased proliferation and EMT, whereas increased apoptosis by targeting MTA1 26.

Villin2 (VIL2), the encoding gene of Ezrin protein, is another downstream target gene of miR-183 that regulates EMT. Ezrin is the earliest member of the ERM (ezrin/radixin/moesin) protein family, which is considered to be an important regulator and connector between cell membrane molecules (such as CD44 and ICAM2) and cytoskeleton (such as actin) 27. It has also been shown that ezrin can coprecipitate with two important adhesion molecules, E-cadherin and beta-catenin, which further illustrates the importance of Ezrin in cell adhesion 28. Overexpression of miR-183 repressed the expression levels of Ezrin and significantly inhibited the motility of osteosarcoma cells via increased N-cadherin and activating ERK signaling 29, 30. MiR-183 was decreased in endometrial cancer tissues, while the expression of Ezrin was significantly increased. The downregulation of miR-183 suppressed apoptosis and promoted EMT of human endometrial cancer cells by upregulating Ezrin 31. In gastric cancer and melanoma, miR-183 acts as a tumor suppressor, the expression was significantly decreased. Overexpression of miR-183 markedly suppressed cell invasion by downregulation of Ezrin expression 32. In breast cancer, miR-183 targets VIL2 and may play a central role in the regulation of metastasis. The above literature indicates that overexpression of miR-183 inhibits the expression of VIL2 gene, thus ultimately inhibiting EMT of cancer cells.

The above evidences suggest that miR-183 participates in the process of EMT by targeting ZEB1, MTA1 and VIL2. Participates in the process of cancer indirectly and lay foundation for the migration and invasion of cancer cells.

### Roles of miR-183 in cell migration and invasion

Numerous studies have shown that miRNAs play an important role in the acquisition of invasive ability and malignant phenotype of cancer cells, including loss of cell adhesion molecules, acquisition of cell motility and proliferation of tumors in target organs. At present, many studies have reported that miR-183 is closely related to the invasion and metastasis of tumors 33. MiR-183 can regulate about 45 tumor-related genes, which are closely related to the adhesion, migration and invasion of various cancer cells. The most studied genes are programmed cell death factor 4 (PDCD4), early growth response one (EGR1) and integrin beta 1 (ITGB1), which play important roles in the migration and invasion of tumors.

PDCD4 is the most studied target gene of miR-183. It has been found that miR-183 acts as an oncogene by targeting PDCD4 in a variety of tumors. Lu et al. found that miR-183 overexpression could promote cell proliferation, inhibit cell apoptosis and decrease G₀/G₁ arrest in SW1990 pancreatic cancer cells by targeting PDCD4 34. In breast cancer, gastric cancer and ESCC, miR-183 is overexpressed, the overexpression significantly enhanced cell proliferation migration, invasion and inhibited cell apoptosis by targeting PDCD4 35, 36, 37, 38. Upregulation of miR-183 is associated with advanced clinical stage, positive lymph node, deep stromal invasion, and distant metastasis in cancer patients 32. Furthermore, knockdown of PDCD4 suppressed expression of p21 and p27 in breast cancer, which shows that miR-183 can inhibit p21 and p27 indirectly 37.

EGR1 plays a very important coupling role in the cascade reaction of information transmission between cells and participates in many life activities such as cell division, proliferation, differentiation and apoptosis. EGR1 can promote apoptosis by activating p53 or binding with transcription factor E-Jun. It has been reported that miR-183 plays an important role in promoting cancer in synovial sarcoma, rhabdomyosarcoma (RMS) and colon cancer by regulating the expression of EGR1 and phosphatase and tensin homolog deleted on chromosome ten (PTEN) 39. Inhibiting or knocking out miR-183 can lead to a significant increase in the expression of EGR1 and PTEN proteins, as well as the effect of inhibiting cell migration and invasion. Thiel J. et al. found that overexpression of miR-183 in murine CD4+ T cells resulted in decreased EGR1 and PTEN expression, elevated Akt phosphorylation and enhanced proliferation 40. In NSCLC, Wang et al. found that miR-183-5p could promote lung carcinogenesis by directly targeting PTEN 41.

ITGB1 plays a key role in migration and invasion of tumors 42. MiR-183 can act as an antioncogene by targeting ITGB1. Many human tumors are related to the increase of ITGB1 expression and activation of ITGB1-related signaling pathways 43. Sun et al. found a new signaling pathway promoting melanoma development by MALAT1-miR-183-ITGB1 axis 13. MiR-183 can significantly reduce the expression of ITGB1, thereby inhibiting cell migration and adhesion. MiR-183 is downregulated in cervical cancer cell line HeLa, and miR-183 directly regulates the invasion and metastasis of HeLa cells by acting on its target gene ITGB1 44.

In addition to the above important genes, there are about thirty miR-183 target genes related to migration and invasion (Table 3). This indicates that miR-183 has a great influence on the invasion and migration of cells. By promoting or inhibiting the migration and invasion of cancer cells, it directly participate the process of cancer.

### Roles of miR-183 in microangiogenesis

Experimental and clinical studies have confirmed that the growth rate of vascularized tumors is significantly faster than that of non-vascularized tumors 45. Hypoxia is a major factor in neovascularization of tumors. It stimulates tumour cells to produce a variety of angiogenic factors, such as vascular endothelial growth factor (VEGF). Fibronectin 1 and serine protease inhibitor family E member 2 (SERPINE2) are essential for vascular mimicry in this system. Low-density lipoprotein receptor-related protein 1 (LRP1) and ITGB1 are receptors for these secreted proteins. Evidence shows that miR-183 regulates those autocrine signal and VM formation 51. In addition, hypoxia-induced transcription factors (HIFs) are upregulated before angiogenesis due to hypoxia. Hypoxia in microenvironment can result in upregulation of growth factor receptors, angiogenic cytokines and proteolytic enzymes 46. Studies in glioma suggest that miR-183 upregulation in malignant gliomas induces HIF-1α expression by targeting IDH2 110. Reducing the translation of miR-183-inhibited HIF-1α and other hypoxic markers can increase angiogenesis and improve oxygen transport in hypoxic tissues.

Effect of miR-183 on human umbilical vascular endothelial cells (HUVECs) and vascular endothelial cells, indirectly showed the role of 183 in angiogenesis. Inhibition miR-183 expression in HUVECs injury could enhance cell activity, inhibit inflammatory level, and thus resist cell injury by upregulating expression of IRS1 47. Overexpression miR-183 promoted cell growth and tube formation while it suppressed cell apoptosis of vascular endothelial cells 48. In addition, miR-183 also participates in microangiogenesis during tumor growth through some signaling pathways. PI3K/AKT/VEGF signaling pathway plays an important role in angiogenesis. Research in diabetic retinopathy revealed the involvement of miR-183 via the PI3K/AKT/VEGF signaling pathway by targeting BTG1. MiR-183 overexpression activated the PI3K/AKT/VEGF signaling pathway and inhibited BTG1 48. These evidences suggest that miR-183 is involved in microangiogenesis through multiple regulations. Angiogenesis plays a decisive role in the growth of tumors and metastasis of cancer cells, which further demonstrates that miR-183 is closely involved in the progression of cancer.

## miR-183 in cancer diagnosis and treatment

In recent decades, the rapid rise of posttranscriptional mechanistic studies, especially miRNA studies, has brought a new perspective to early diagnosis and precise treatment of tumors. Because the expression of miRNA in blood is relatively stable, the content of miRNA in serum can provide important information for the prediction and diagnosis of cancer. Compared with the level of expression of mRNA and protein, miRNA will not face the fate of being regulated and degraded, so its detection results are relatively more reliable. MiR-183 is highly abnormally expressed in cancer patients and easy to detect. Therefore, on the basis of understanding miR-183 and its mechanism, the expression of miR-183 in blood can be used to diagnose cancer and determine the appropriate treatment strategies.

Targeting drug brings some diagnostic advantages. However, it does not work for a considerable number of patients, or some of the active patients develop drug resistance after a period of treatment, leading to further deterioration of the condition and increasing the difficulty of diagnosis. A study found that miR-183 may be involved in paclitaxel-induced chemotherapy when studying the miRNA related to paclitaxel-induced apoptosis of hepatocellular carcinoma 110. Abnormal expression of miR-183 in ovarian cancer cells also results in chemical resistance to paclitaxel 111. It has been reported that miR-183 regulates multidrug resistance (MDR) in hepatocellular carcinoma through a feedback loop of miR-183 idh2/SOCS6-HIF-1α axis 112. MiR-183 has been up-regulated in MDR Ehrlich ascites tumor cells and regulated MDR in hepatocellular carcinoma cells. Kouri et al. synthesized spherical nucleic acid based on functionalized gold nanoparticles with mature miR-182 double chains could reduce the burden of tumors and increase the survival rate of animals through intravenous application 113, which can also use anti-miR-183 as a possible treatment in cancer. Notably, miR-183 might affect the prediction for PSA-dependent diagnosis and prognosis via regulating PSA expression 100. However, there are many kinds of miRNA involved in this process. Therefore, further research is needed to thoroughly investigate how complex miRNA regulatory networks contribute to the MDR process 114. Study of the specific regulatory mechanisms of miR-183 in these tumors can further elucidate the molecular mechanisms of tumorigenesis and development from the perspective of genes, which will form a complete molecular signaling pathway and functional gene network system related to miR-183. This provides an important guiding value for the treatment of clinical tumors, and also makes the early diagnosis and precise treatment of tumors possible.

## Discussion

**Figure 2:**
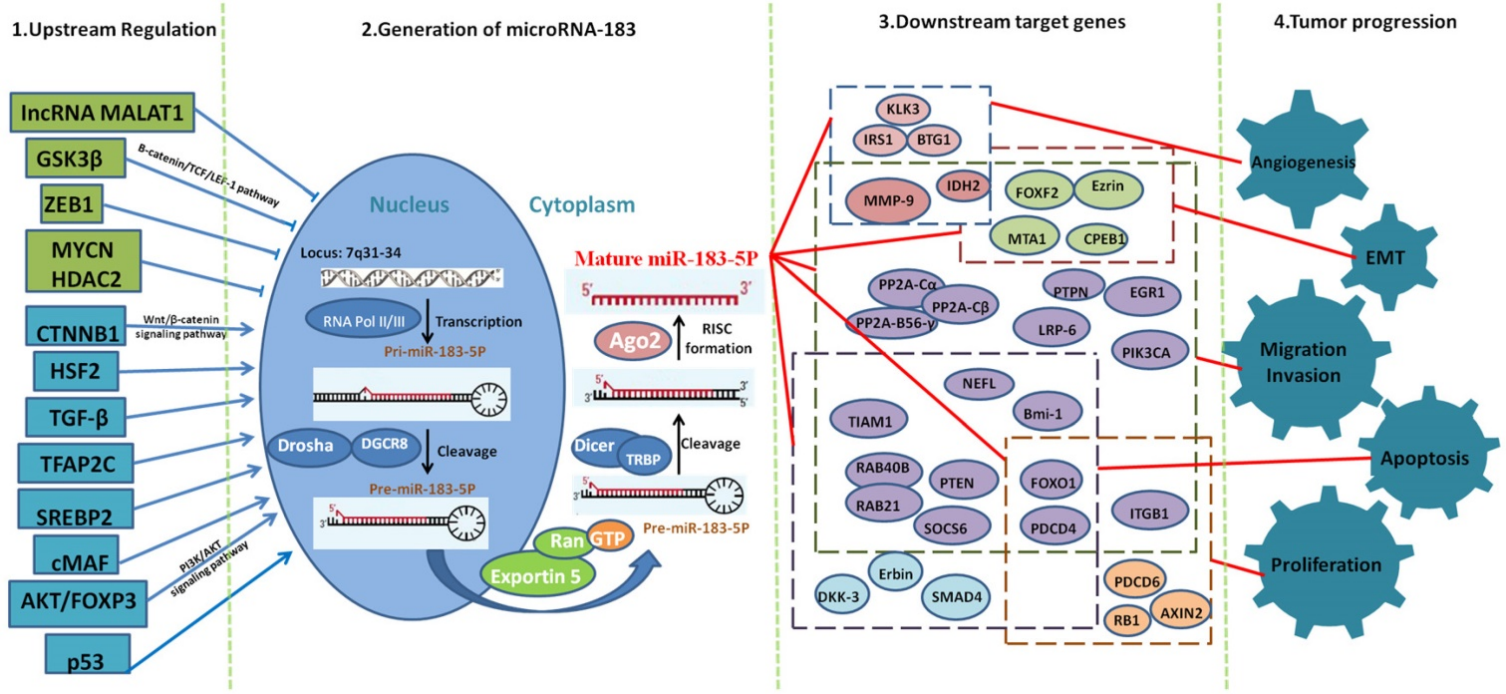
Mechanisms of miR-183 in cancer progression at cellular and molecular level.

The recognized mechanism of miRNAs is to influence the stability of the target gene by complementary or incomplete pairing, inhibit its translation and ultimately achieve the regulation of protein expression 49. Previous studies have suggested that miRNAs play a role only in complete or incomplete pairing with the 3'-UTR region of mRNA. Studies have shown that it can also play a role in combination with the coding region of mRNAs and the 5'UTR region 50. Therefore, miR-183 may have more target genes and play more important roles in the biological process, especially in cell proliferation, migration, invasion and EMT (Fig. 2). Many biological processes, such as growth, development, embryo implantation and embryo development, all involve cell proliferation, migration, invasion, and EMT. This indicates that miR-183 may play a much more important role in biological physiological regulation.

While in cancer, the change pattern of miR-183 varies greatly among different types of cancer. Different upstream regulators play a key role in the expression of miR-183 in different cancer types. We can conclude that miR-183 is upregulated in most cancer types. Meanwhile, up to now, 8 upstream regulators can upregulate the expression of miR-183 and 5 upstream regulators can downregulate the expression of miR-183. We speculate that miR-183 is upregulated in most cancer types because of the fact that there are more upregulating effect transcription factors than downregulating effect transcription factors.

It has been reported that miR-183 plays different roles in cell migration and invasion in different tissues. We speculate that the main reason for this result is that a miRNA can have hundreds of target genes. Through network miRNAs target gene prediction system, we found that the potential downstream targets of miR-183 include oncogenes, antioncogenes, cell signal transduction molecules, cell cycle regulation genes and molecules related to invasion and metastasis. As far as the mechanism of miRNAs is concerned, miR-183 can play the role of antioncogene by inhibiting the expression of oncogene, inhibiting cell migration and invasion. Of course, it can also play the role of oncogene by inhibiting the expression of antioncogene, promoting cell migration and invasion. This suggests that miR-183 plays different roles in different tumors, and its molecular mechanisms are complex and diverse, which need to be further explored. In addition, the abnormal expression of miR-183 in cancer progression can be used as a biological molecule for cancer diagnosis and treatment; it has great significance for cancer diagnosis and development of new therapeutic drugs.

## Figures and Tables

**Table 1:** Upstream regulator of miR-183 expression.

Upstream regulator	Model system	Direction of regulation	Source
IncRNA MALAT1	Melanoma	↓	Sun Y, et al.(2017) 13
ZEB1	breast cancer	↓	Langer EM, et al.(2018) 50
			Li P, et al.(2014) 10
GSK3β	gastric cancer	↓	Tang X, et al.(2013) 8
MYCN, HDAC2	Neuroblastoma	↓	Lodrini M, et al.(2013) 11
CTNNB1	hepatocellular carcinoma	↑	Leung WK, et al.(2015) 7
HSF2	breast cancer	↑	Li P, et al.(2014) 10
TGF-β	lung tumor	↑	Trinh TL, et al.(2019) 52
AKT/FOXP3	leukemia U937 cells	↑	Liu W, et al.(2012) 53
TFAP2C	non-small cell lung cancer	↑	Kang J, et al.(2017) 54
SREBP2	Murine liver	↑	Jeon T, et al.(2013) 55
cMAF	Murine liver	↑	Kelada S, et al.(2013) 56
p53	Primary human mammary epithelial cells	↑	Chang C, et al.(2011) 57

**Table 2:** Up and down regulation of miR-183 expression in different cancer in published articles.

Cancer type	Regulated features		Source
Hepatocellular carcinoma	↕	upregulated	Bharali D, et al.(2018) 58
			Li ZB, et al.(2015) 59
			Leung WK, et al.(2015) 7
			Li J, et al.(2010) 14
		downregulated	Bian W, et al.(2018) 60
Endometrial cancer	↕	upregulated	Ruan H, et al.(2017) 61
			Xiong H, et al.(2018) 23
		downregulated	Yan H, et al.(2018) 31
Non-small cell lung cancer	↕	upregulated	Wang H, et al.(2019) 41
			Zhang L, et al.(2015) 62
		downregulated	Yang C, et al.(2018) 63
			Yang X, et al.(2018) 64
Gastric cancer	↕	upregulated	Li C, et al.(2016) 35
			Gu W, et al.(2014) 32
		downregulated	Cao LL, et al.(2014) 65
			Xu L, et al.(2014) 66
Cervical cancer	↕	upregulated	Liu SS, et al.(2018) 67
		downregulated	Zhang W, et al.(2018) 44
			Fan D, et al.(2016) 68
Lung cancer	↕	upregulated	Trinh TL, et al.(2019) 52
		downregulated	Meng F, et al.(2019) 69
Pancreatic adenocarcinoma	↕	upregulated	Miao F, et al.(2016) 70
		downregulated	Zhou L, et al.(2014) 71
Prostate cancer	↑	upregulated	Waseem M, et al.(2019) 72
			Ueno K, et al.(2013) 73
Ovarian cancer	↑	upregulated	Zhou J, et al.(2019) 74
Esophageal squamous cell carcinoma	↑	upregulated	Yang M, et al.(2014) 36
			Ren L, et al.(2014) 12
Mesothelioma	↑	upregulated	Suzuki R, et al.(2018) 75
Bladder cancer	↑	upregulated	Chen D, et al.(2018) 76
Renal cell carcinoma	↑	upregulated	Zhang XL, et al.(2018) 77
			Qiu M, et al.(2014) 78
Colorectal cancer	↑	upregulated	Chen Y, et al.(2018) 79
			Yuan D, et al.(2015) 80
Tongue carcinoma	↑	upregulated	Supic G, et al.(2018) 81
Tongue squamous cell carcinoma	↑	upregulated	Yan D, et al.(2016) 82
Pediatric caute myeloid leukemia	↑	upregulated	Wang X, et al.(2017) 9
Breast cancer	↑	upregulated	Cheng Y, et al.(2016) 37
			Li P, et al.(2014) 10
Brain glioma	↑	upregulated	Ye Z, et al.(2016) 83
			Wang Z, et al.(2016) 84
			Tanaka H, et al.(2013) 109
Epithelial ovarian cancer	↑	upregulated	Chen H, et al.(2016) 85
Vulvar squamous cell carcinoma	↑	upregulated	Yang X, et al.(2016) 86
Papillary thyroid carcinoma	↑	upregulated	Wei C, et al.(2015) 38
Synovial sarcoma	↑	upregulated	Sarver AL, et al.(2010) 39
Rhabdomyosarcoma (RMS)	↑	upregulated	Sarver AL, et al. (2010) 39
Colon cancer	↑	upregulated	Sarver AL, et al. (2010) 39
Osteosarcoma	↓	downregulated	Sun X, et al.(2018) 24
			Zhang J, et al.(2014) 30
Nasopharyngeal carcinoma	↓	downregulated	Wang G, et al.(2017) 25
			Cheung CC, et al.(2016) 87
Melanoma	↓	downregulated	Sun Y, et al.(2017) 13
Retinoblastoma	↓	downregulated	Wang J, et al.(2014) 88
Kidney renal clear cell carcinoma	↓	downregulated	Yuan J, et al.(2019) 89
Infected abdominal aortic aneurysm	↓	downregulated	Meng C, et al.(2018) 90

**Table 3:** Genes that have been shown to have a targeting relationship with miR-183 and their functions in the corresponding cancer types or cell lines.

Model system	Tissue (specimens)	Cell lines	Target gene	Function	Source
pancreatic cancer	X	SW1990 Pancreatic cancer cell line	PDCD4	Apoptosis, Proliferation	Lu Y, et al.(2016) 34
breast cancer	√ (n=18)	MCF-7 and MDAMB-231	PDCD4	Apoptosis, Proliferation	Cheng Y, et al.(2016) 37
esophageal squamous cell carcinoma	√ (n=81)	EC109 and EC9706	PDCD4	Apoptosis, Proliferation	Yang M, et al.(2014) 36
gastric cancer	√ (n=80)	SGC-7901	PDCD4	Apoptosis, Proliferation	Gu W, et al.(2014) 32
papillary thyroid carcinoma	√ (n=38)	TPC-1, BCPAP, K1 and NPA PTC	PDCD4	Migration, Apoptosis	Wei C, et al.(2015) 38
hepatocellular carcinoma	√ (n=25)	HepG2 and Huh7	PDCD4	Apoptosis	Li J, et al.(2010) 14
esophageal squamous cell carcinoma	√ (n=32)	Eca109 and TE13	PDCD4	Proliferation, Invasion	Ren L, et al.(2014) 12
pediatric caute myeloid leukemia	√ (n=106)	HL60 and K562	PDCD6	Apoptosis	Wang X, et al.(2017) 9
melanoma	X	A375 human melanoma cells	MMP-9	Migration, Invasion	Zhang Y, et al.(2019) 91
endometrial cancer	√ (n=30)	KLE, HEC-1-A and HHUA	MMP-9	Proliferation, Invasion	Ruan H, et al.(2017) 61
cervical cancer	√ (n=53)	siha and Hela	MMP-9	Invasion, Metastasis	Fan D, et al.(2016) 68
osteosarcoma	√ (n=25)	hFOB 1.19 and MG63	MTA1	Migration, Invasion	Sun X, et al.(2018) 24
nasopharyngeal carcinoma	√ (n=29)	C666-1, CNE1, CNE2, HONE1, and 5-8F	MTA1	Apoptosis	Wang G, et al.(2017) 25
hepatocellular carcinoma	√ (n=10)	HepG2	MTA1	EMT, Metastasis	Bian W, et al.(2018) 60
non-small cell lung cancer	√ (n=194)	H1299, SPC-A-1, 95D and A549	MTA1	Migration, Invasion	Yang C, et al.(2018) 63
pancreatic cancer	√ (n=108)	SW1900 Pancreatic cancer cell line	MTA1	EMT, Migration, Invasion	Lin X, et al.(2017) 26
osteosarcoma	X	F5M2 and F4	VIL2(Ezrin)	Invasion	Zhao H, et al.(2012) 29
melanoma	X	A375 human melanoma cells	VIL2(Ezrin)	Migration, Invasion	Zhang Y, et al.(2019) 91
endometrial cancer	√ (n=156)	Ishikawa,KLE, JEc, HEc-1-A, and HHUA	VIL2(Ezrin)	EMT, Migration, Invasion	Yan H, et al.(2018) 31
gastric cancer	√ (n=55)	MGC-803, SGC-7901,BGC-823, MKN-45, and MKN-28	VIL2(Ezrin)	Invasion	Cao LL, et al.(2014) 65
lung cancer cells	X	801D and 95C	VIL2(Ezrin)	Migration, Invasion	Wang G, et al.(2008) 92
breast cancer	√ (n=70)	MDA-MB-231,T47D,ZR-75-1,and SKBR-3	VIL2(Ezrin)	Migration	Lowery AJ, et al.(2010) 93
acute myeloid leukemia	√ (n=7)	HL-60, U937	Erbin	Proliferation	Zheng Z, et al.(2019) 94
pancreatic adenocarcinoma	√ (n=52)	PANC-1 and HPDE6‑C7	SOCS6	Proliferation, Invasion,	Miao F, et al.(2016) 70
hepatocellular carcinoma	√ (n=10)	HepG2 and Hep3B	SOCS6	Growth, Invasion	Li ZB, et al.(2015) 59
cervical cancer	√ (n=43)	SiHa, C-33 A, C-4-I, and CaSki	ITGB1	Migration, Invasion	Zhang W, et al.(2018) 44
melanoma	√ (n=30)	HBL and SK-MEL-1	ITGB1	Tumor progression	Sun Y, et al.(2017) 13
HeLa cells	X	HeLa,HDF and HTM	ITGB1	Migration, Invasion	Li G, et al.(2010) 95
gastric cancer	X	MKN28	UVRAG	Autophagosomes ,UVR	Yuan Y, et al.(2018) 96
colorectal cancer	√ (n=14)	HCT116 and HT29	UVRAG	Autophagosomes, UVR	Huangfu L, et al.(2015) 97
mesothelioma	√ (n=29)	ACC-MESO1 and CRL-5915	FOXO1	Migration, Invasion	Suzuki R, et al.(2018) 75
lymphoma	-	L428	FOXO1	Proliferation	Xie L, et al.(2012) 98
breast cancer	X	MCF-7	RAD50	DNA repair processes	Shirode AB, et al.(2014) 99
prostate cancer	√ (n=49)	DU145, PC3, 22Rv1 and LNCaP-ARhi	KLK3, PSA	Growth	Larne O, et al.(2015) 100
ovarian cancer	√ (n=30)	SKOV-3, OVcAR3 and HOSE	SMAD4	Apoptosis, Proliferation	Zhou J, et al.(2019) 74
prostate cancer	√ (n=31)	PC-3, DU-145 and LNCaP	SMAD4	Growth	Ueno K, et al.(2013) 73
lung tumor	√ (n=126)	293T cells, H1355 and H1299	MICA,MICB	Immune correlation	Trinh TL, et al.(2019) 52
pancreatic ductal adenocarcinoma	√ (n=91)	ASPC-1, SW1990, BXPC-3, CFPAC-1 and PANC-1	Bmi-1	Proliferation	Zhou L, et al.(2014) 71
gastric cancer	√ (n=65)	AGS, SGC7901, MKN28, MGC803 and HGC27	Bmi-1	Proliferation, Invasion	Xu L, et al.(2014) 66
osteosarcoma	X	MG63 and U20S	LRP-6	Invasion	Yang X, et al.(2018) 64
retinoblastoma	√ (n=6)	Y79, SO-RB50 and WERI-RB1	LRP-6	Growth, Invasion	Wang J, et al.(2014) 88
synovial sarcoma	√ (n>300)	SYO-1 and FUJI	EGR1	Migration	Sarver AL, et al.(2010)^39^
colon cancer	√ (n=80)	HCT116 and DLD1			
rhabdomyosarcoma	√ (n=42)	Rh30 and JR1			
non-small cell lung cancer	√ (n=55)	A549, SPCA-1, PC-9, and 95-D	PTEN	Tumor progression	Wang H, et al.(2019) 41
leukemia	X	leukemia U937 cells	ADAM17, NFK-β	--	Liu W, et al.(2012) 53
renal cell carcinoma	X	ACHN, 786-O, Caki-1 and Caki-2	DKK-3	Proliferation, Metastasis	Zhang XL, et al.(2018) 77
prostate cancer	√ (n=31)	PC-3, DU-145 and LNCaP	DKK-3	Growth	Ueno K, et al.(2013) 73
gastric cancer	√ (n=876)	AGS and MKN-45	TCF12	Tumor progression	Wang X, et al.(2019) 101
lung cancer	√ (n=15)	A549, 95D, H1299 and H1650	PIK3CA	Migration, Invasion	Meng F, et al.(2019) 69
bladder cancer	√ (n=23)	5637 and T24	AXIN2	Growth, Apoptosis	Chen D, et al.(2018) 76
endometrial cancer	√ (n=208)	Ishikawa and RL95-2	CPEB1	EMT, Migration, Invasion	Xiong H, et al.(2018) 23
glioblastoma	√ (n=39)	U251R	LRIG1	Radioresistance	Fan H, et al.(2018) 102
breast cancer cell line	X	MDA-MB-231 and MDA-MB-468	RB1	Metastasis	Macedo T, et al.(2017) 103
lung adenocarcinoma	√	CSLCs	PTPN	Migration, Invasion	Zhu C, et al.(2016) 104
colon cancer	√ (n=10)	DLD1, Caco-2, HCT116, DiFi, and HCA7	ABCA1	Apoptosis, Proliferation	Bi DP, et al.(2016) 105
glioma	√ (n=44)	U87MG, U251, and LN229	NEFL	Proliferation, Invasion	Wang Z, et al.(2016) 84
lung cancer	X	344SQ, 344LN, 531LN1 and 531LN2	FOXF2	EMT, invasion	Kundu ST, et al.(2016) 22
breast cancer	√ (n=2)	MCF-7,MDA-MB-231 and MCF-10A et al.	RAB40B ,RAB21	Proliferation, Migration	Li P, et al.(2014) 10
renal cancer cells	√ (n=16)	ACHN and A498	PP2A-Cα, PP2A-Cβ, PP2A-B56-γ	Migration, Invasion	Qiu M, et al.(2014) 78
glioma	√ (n=88)	U251, U87MG, T98G, A172 and SF126	IDH2	Angiogenesis	Tanaka H, et al.(2013) 109
ovarian cancer	√ (n=17)	SKOV-3ip and HO-8910PM	TIAM1	Migration, Invasion	Li J, et al.(2012) 106
HeLa cells	X	HeLa	KIF2A	Migration, Invasion	Li G, et al.(2010) 95
HUVECs	X	HUVECs	IRS1	Angiogenesis	Zhang Y, et al.(2019) 47
diabetic retinopahy	X		BTG1	Angiogenesis	Zhang ZZ, et al.(2019) 107
			NF-KB1	--	Sha F, et al.(2014) 108

X: No relevant data or use of pathological tissue for testing. √: Pathological tissue tests were carried out.
